# A family of killers

**DOI:** 10.7554/eLife.49211

**Published:** 2019-07-26

**Authors:** Mickaël De Carvalho, Sarah E Zanders

**Affiliations:** 1 Stowers Institute for Medical ResearchKansas CityUnited States; 2 Open UniversityMilton KeynesUnited Kingdom; 3 Department of Molecular and Integrative PhysiologyUniversity of Kansas Medical CenterKansas CityUnited States

**Keywords:** *Podospora*, selfish genetic element, spore killer, genomic conflict, fungi, gene drive, Other

## Abstract

*Spok* genes are meiotic drivers that increase their own chances of transmission by killing gametes that do not inherit them.

**Related research article** Vogan AA, Ament-Velásquez SL, Granger-Farbos A, Svedberg J, Bastiaans E, Debets AJM, Coustou V, Yvanne H, Clavé C, Saupe SJ, Johannesson H. 2019. Combinations of *Spok* genes create multiple meiotic drivers in *Podospora*. *eLife* **8**:e46454. doi: 10.7554/eLife.46454

Some genes are born criminals. Mendel’s law of segregation states that the two alleles of a heterozygote are each transmitted into half of the gametes, but genes called meiotic drivers break this law by striving to be the only gene variant inherited. This selfish behavior can help them to spread in a population, but it can also decrease the fitness of the organisms carrying the loci (Sandler and Novitski, 1957; Zimmering et al., 1970; Zanders and Unckless, 2019).

Meiotic drivers employ a variety of self-promoting mechanisms (Lindholm et al., 2016). Some are particularly ruthless because they kill the gametes or spores that do not inherit the drive gene. Meiotic drivers are found throughout eukaryotes, including mammals, but relatively little is known about the genes responsible. Now, in eLife, Hanna Johannesson and co-workers at Uppsala University, the University of Bordeaux and Wageningen University – including Aaron Vogan and Lorena Ament-Velásquez, both from Uppsala, as joint first authors – report on the genetic architecture underlying a series of killer meiotic drive phenotypes in the fungus *Podospora anserina* (Vogan et al., 2019).

*P. anserina* is an excellent model organism for studying meiotic drive, largely because the phenotype is easy to observe: the two spores that inherit the drive locus are viable and pigmented, while the two spores that do not are dead and unpigmented (Figure 1A; Padieu and Bernet, 1967; Turner and Perkins, 1991; Espagne et al., 2008; Dalstra et al., 2003).

**Figure 1.**
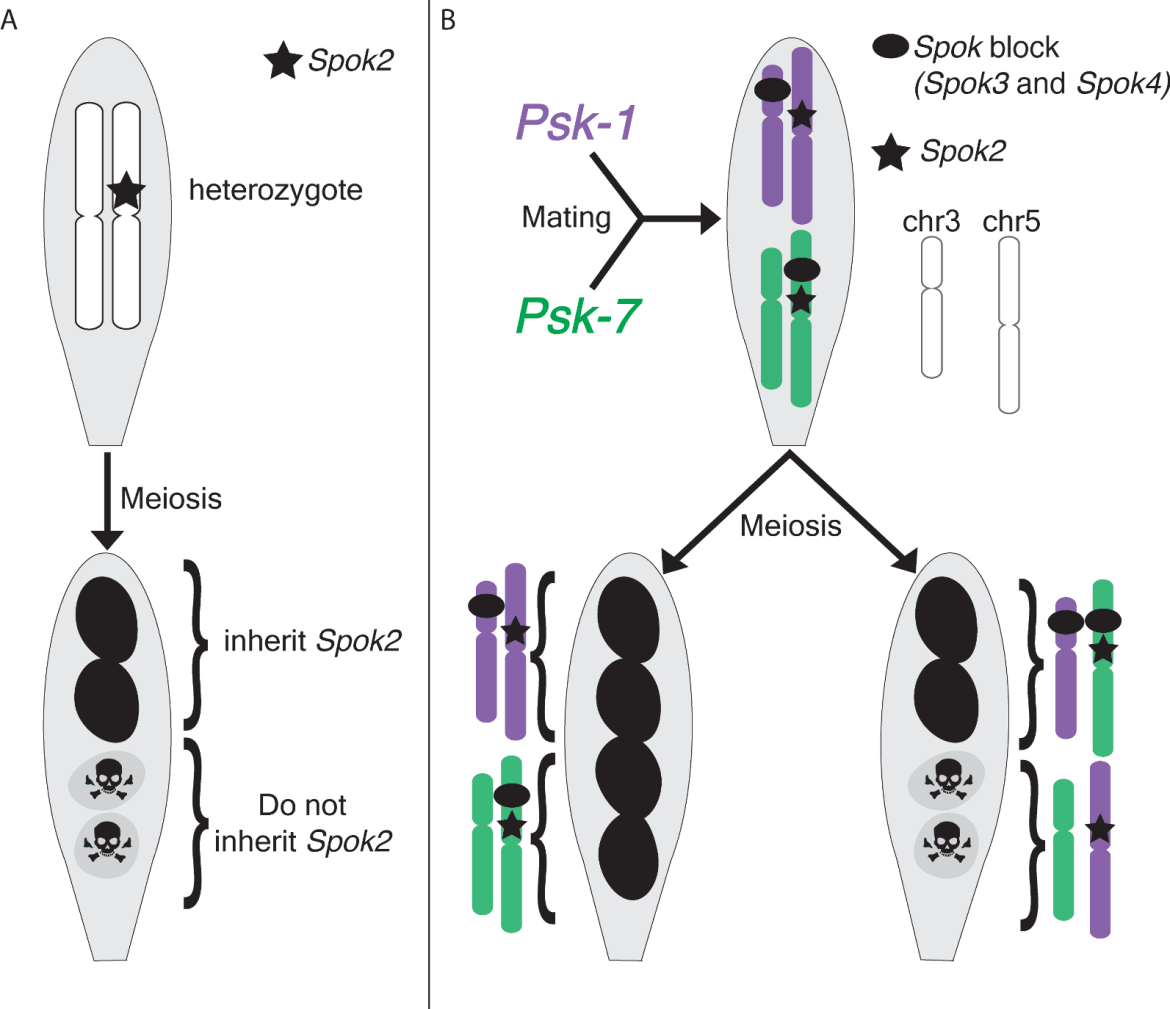
Meiotic drive in *Podospora anserina*. (**A**) A heterozygous diploid generated by mating a naïve strain to one carrying *Spok2* undergoes meiosis and generates four spores. One possible outcome of meiosis is depicted. The spores that inherit *Spok2* are alive (pigmented), while the spores that do not inherit the *Spok2* gene are destroyed (unpigmented, hazard signs). (**B**) A heterozygous diploid generated by mating the *Psk-1* strain (purple) to the *Psk-7* strain (green) undergoes meiosis and generates four spores. Note that the *Spok* block (black oval) is on different chromosomes in the two isolates. Two possible outcomes are depicted: (left) all four spores survive because they all inherited *Spok2* and the block that contains *Spok3* and *Spok4*; (right) two of the four spores do not survive because they did not inherit the *Spok* block.

*P. anserina* strains carrying spore-killing loci have been sorted into seven types (*Psks*) based on their killing phenotypes, but the genes underlying these phenotypes were unknown (van der Gaag et al., 2000). Recently, *Spok2* was identified as an autonomous single-gene meiotic driver in *P. anserina*, and it was shown that the *Spok* genes comprise a gene family found in many copies in diverse fungal lineages (Grognet et al., 2014). These discoveries suggested the *Spok* genes as candidates for causing the *Psk* phenotypes.

Vogan et al. started by sequencing and assembling the genomes of six *P. anserina* strains representing the distinct spore-killing phenotypes. These assemblies showed that *Spok2* is present in most *P. anserina* isolates and facilitated the discovery of two new *Spok* genes (*Spok*3 and *Spok4*) that are both spore killers. Curiously, these two genes are found within a block of sequence that is found in single copy at different locations within the various genomes. In some, the block contains only *Spok3* or *Spok4*, but in others it contains both. How the block has moved during the evolutionary history of *P. anserina* is a mystery.

Next, Vogan et al. complemented their genomic approach with classical genetic analyses. They demonstrated that all the *Psk* phenotypes can be explained by just three genes (*Spok2*, *Spok3* and *Spok4*). Spore killing occurs when two out of the four spores fail to inherit one or more of the *Spok* genes present in the diploid cell that underwent meiosis (Figure 1A and B). Strains with no *Spok* genes are considered naïve and spore killing will occur when these strains are crossed to *Psk* strains, but not to other naïve strains. The most dominant *Psk* phenotypes, *Psk-1* and *Psk-7*, contain all three *Spok* genes. These strains cause killing when crossed to all other types. Interestingly, killing is also observed when *Psk-1* and *Psk-7* are crossed to each other because the *Spok* block is found at different loci in the two strains, which means that some spores do not inherit it (Figure 1B). The other strains that cause killing contain one or two *Spok* genes. Again, their phenotypes are explained by the identity and location of their *Spok* genes.

The latest work also provides a significant advance in our understanding of the mechanisms used by *Spok* genes. Previous work demonstrated that these genes encode both killing and resistance functions in genetically separable domains and predicted a kinase domain in one of these regions (Grognet et al., 2014). The new work shows that *Spok3* can be expressed outside of spore formation, and Vogan et al. exploit this observation to better characterize how single *Spok* genes carry out their dual roles. In particular they show that the region required for the resistance function must include a cysteine-rich domain (in addition to the kinase domain mentioned above). Moreover, they identify a nuclease domain and demonstrate that it is required for the killing function. However, how the nuclease domain promotes killing and how the kinase domain neutralizes that killing are unknown.

It is becoming clear that genetic criminals that break Mendel’s law of segregation are not rare. Discovering the mechanisms used by meiotic drive genes and how they evolve is necessary to fully understand gametogenesis. Exciting discoveries in *Podospora* will continue to shape our understanding of the molecular genetics of meiotic drive.
